# Editorial: Advances in limb-salvage surgery and reconstruction for pediatric bone and soft tissue tumors

**DOI:** 10.3389/fped.2026.1817788

**Published:** 2026-04-17

**Authors:** Hisaki Aiba, Makoto Yamaguchi, Hiroaki Kimura, Hideki Murakami

**Affiliations:** Department of Orthopaedic Surgery, Nagoya City University Graduate School of Medical Sciences, Nagoya, Japan

**Keywords:** aneurysmal bone cyst, chodroblastoma, Masquelet technique, minimally invasive surgery, simple bone cyst, surgical hip dislocation

## 1 Introduction

Recent technological and multidisciplinary advances have markedly expanded limb-salvage options for pediatric bone and soft tissue tumors, reducing the need for amputation while improving functionality. This editorial reviewed the current special issue, “*Advances in Limb-Salvage Surgery and Reconstruction for Pediatric Bone and Soft Tissue Tumors*”, and focused on the treatment of benign bone tumors.

## 2 Percutaneous intramedullary aspiration, irrigation, and injection of absorbable bone (PAIB) technique

Simple bone cyst (SBC) is a benign bone tumor in children and compromises bone integrity by reducing cortical thickness and causing cortical expansion, thereby increasing the risk of pathological fractures (1, 2). Among the various surgical interventions, percutaneous intramedullary aspiration, irrigation, and injection of absorbable bone (PAIB) is a minimally invasive option for the treatment of SBC. PAIB includes the following steps: 1. Percutaneous punctures to the tumor at both ends; 2. Irrigation of the intramedullary cavity with a large volume of saline; and 3. Injection of absorbable artificial bone graft material into the cavity (Xu et al.).

In a retrospective cohort of 36 children with SBC who underwent PAIB between 2019 and 2024, Xu et al. reported that recurrence occurred in six patients (16.6%). Five of the recurrent cases underwent repeat PAIB procedures, resulting in complete resolution in two patients. Bone healing was eventually achieved in 32 patients (88.9%). No cases of limb dysfunction, nerve injury, heterotopic ossification, surgical site infection, or epiphyseal damage were observed at the final follow-up (Xu et al.).

## 3 Curettage and bone grafting combined with electrocautery and drill, supplemented with plate fixation technique

Aneurysmal bone cyst (ABC) predominantly affects children and is pathologically characterized by blood-filled cystic cavities surrounded by fibrous tissue and multinucleated giant cells (3). It can cause localized pain, swelling, limited mobility, and pathological fractures (4). For skeletally immature children, surgical intervention for ABC should balance between complete lesion removal and growth plate preservation (5). The combination of curettage, bone grafting, electrocautery and drill, and plate fixation could be a promising approach since the use of an electrocautery and drill allows for more thorough lesion removal, bone grafting fills defects and promotes bone healing, and the plate fixation provides mechanical stability (Liang et al.).

In a retrospective study of 23 patients with humeral ABC who underwent this procedure, Liang et al. analyzed postoperative Visual Analog Scale (VAS) function via the Constant–Murley score as well as radiographic healing and recurrence. VAS decreased significantly from a preoperative average of 5.6–1.1 at 1-year post procedure (*P* < 0.05). Constant–Murley score improved from a preoperative average of 42–87 at 1-year post procedure (*P* < 0.05). Radiographic evaluation confirmed lesion clearance and bone fusion in all patients, with no recurrence during follow-up (Liang et al.).

## 4 Direct anterior approach for femoral head with surgical hip dislocation (DAA-SHD) technique

Chondroblastoma typically arises in the epiphyses or apophyses of long bones, and the femoral head is challenging site due to approach (6). The traditional surgical approach for the femoral head can risk physeal injury and avascular necrosis (7).

The direct anterior approach for femoral head with surgical hip dislocation (DAA-SHD) technique is performed with the patient in a supine position (Yang et al.). A direct anterior incision is made in the Hueter interval between the tensor fasciae latae and sartorius muscles, and the plane between rectus femoris and gluteus medius is developed to expose the anterior hip capsule. With a T-shaped capsulotomy, the ligamentum teres is released and the femoral head is anteriorly dislocated. The tumor is carefully curetted, and the cavity is irrigated with sterile water and ethanol. The defect is grafted with a mixture of autologous iliac cancellous bone, allograft bone fragments, calcium sulfate, and osteoinductive materials. An autologous iliac cortical graft is used to reconstruct the articular surface.

In a retrospective analysis of four cases treated with DAA-SHD and autologous iliac bone grafting between 2014 and 2025, Yang et al. assessed hip function via the Modified Injury Severity Scale (MSTS) scale. They reported excellent/good MSTS scores (25–29 points) in all four cases; imaging showed satisfactory graft healing, with no avascular necrosis or recurrence (Yang et al.).

## 5 Two-stage operation of fibula transplantation using the induced membrane (Masquelet) technique

For the treatment of bone defects, vascularized bone grafting, non-vascularized bone grafting, or the Ilizarov technique can be used. However, when the bone defect exceeds 5 cm, the risk of absorption of the non-vascularized bone graft increases (8). An induced membrane (Masquelet) technique is used to promote the engraftment of autologous bone graft (9): the first stage of surgery involves resection of the tumor and filling the bone cavity with bone cement and the second stage involves removal of the bone cement, autogenous fibular bone grafting, internal fixation, and plaster-cast immobilization.

Li et al. reported the two-stage Masquelet technique for chondroblastoma of the first metatarsal in a 9-year-old girl. Four months postoperatively, the metatarsal bone had healed well, the child could walk normally, and no recurrence was observed at 1-year follow-up (Li et al.).

## 6 Conclusion

Various surgical techniques have been attempted for benign tumors in children because of concerns about damage to the epiphyseal plate, growth disturbance or deformity, difficult anatomical access, and morphological differences from adults. Although several novel techniques were proposed in this special issue, their effectiveness should be evaluated in large-scale studies.
